# Sex‐Dependent Effects of Prenatal Stress on Social Memory in Rats: A Role for Differential Expression of Central Vasopressin‐1a Receptors

**DOI:** 10.1111/jne.12343

**Published:** 2016-04-25

**Authors:** N. J. Grundwald, D. P. Benítez, P. J. Brunton

**Affiliations:** ^1^ Division of NeurobiologyThe Roslin Institute and R(D)SVSUniversity of EdinburghMidlothianUK

**Keywords:** bed nucleus stria terminalis, lateral septum, oxytocin receptor, prenatal stress, social recognition, vasopressin‐1a receptor

## Abstract

Prenatal stress (PNS) affects a number of traits in the offspring, including stress axis regulation, emotionality and cognition; however, much less is known about the effects of PNS on social memory and the underlying central mechanisms. In the present study, we investigated social preference, social memory under basal and stress conditions and olfactory memory for social and nonsocial odours in the adult offspring of dams exposed to social stress during late pregnancy. Given the key roles that the central oxytocin and vasopressin systems play in facilitating social memory, we further investigated the effects of PNS on the central expression of mRNA for oxytocin (*Oxtr*) and vasopressin‐1a (*Avpr1a*) receptors. PNS did not affect social preference in either sex; however, social memory was impaired under basal conditions in PNS females but not PNS males. Accordingly, *Avpr1a* mRNA expression in the lateral septum and bed nucleus of stria terminalis (BNST) was unaltered in males but was significantly lower in PNS females compared to controls. No differences in *Oxtr* mRNA expression were detected between control and PNS offspring in either sex in any of the brain regions examined. Social memory deficits in PNS females persisted when social odours were used; however, this does not appear to be a result of impaired olfaction because memory for nonsocial odours was similar in control and PNS females. Under acute stress conditions, deficits in social memory were observed in both male and female control offspring; however, PNS males were unaffected. Moreover, acute stress facilitated social memory in PNS females and this was associated with an up‐regulation of *Avpr1a* mRNA in the lateral septum and BNST. Our data support a role for altered signalling via central Avpr1a in PNS‐induced sex‐dependent changes in social memory and may have implications for understanding the aetiology of neurodevelopmental disorders characterised by social behaviour deficits in humans.

The prenatal period is a time of active neural plasticity and, as such, environmental perturbations during this time can re‐programme brain development. Adverse experiences during development, such as prenatal stress exposure, can have long‐term programming effects on the brain and influence physiology and behaviour in later life 1. Dysregulation of the neuroendocrine stress axis and increased anxiety‐like behaviours are commonly observed phenotypes in prenatally stressed (PNS) rodents and humans 2, 3, 4, 5. Moreover, there is evidence to suggest that prenatal stress also negatively affects aspects of cognitive function, such as spatial, working and reference memory, and object recognition 6, 7, 8. However, much less is known about the impact of prenatal stress on social memory 9.

Social recognition is a fundamental prerequisite for establishing and maintaining social relationships, which serve to maximise both individual survival and successful reproduction of the species. For example, partner‐bonding, parent–offspring bonding and the formation of social dominance hierarchies all rely on the capacity of an animal to remember a familiar conspecific by forming social memories. Social memory is distinct from spatial memory or object recognition and is regulated by different mechanisms and neural circuitry 10. In many mammalian species, centrally acting oxytocin and arginine vasopressin (AVP), together with their receptors, play a crucial role in the neural processing of olfactory signals used for effective social recognition 11, 12.

Oxytocin and AVP are closely‐related peptides (differing by only two amino acids) that are primarily synthesised by neurones located in the supraoptic (SON) and paraventricular nuclei (PVN) of the hypothalamus 13. In the periphery, oxytocin acts to facilitate parturition and is essential in the milk‐ejection reflex during lactation, whereas AVP acts on the kidney to promote water reabsorption 13. Besides these well‐established roles in the periphery, animal studies have amply demonstrated that oxytocin and AVP play pivotal roles in the brain in mediating complex social behaviours, including sexual behaviour, maternal behaviour, mother–offspring bonding and partner‐bonding, as well as social recognition 11, 14, 15, 16, 17. Moreover, emerging evidence implicates the dysfunction of the oxytocin and AVP system in human disorders characterised by social deficits such as autism, social anxiety disorder and schizophrenia, prompting considerable research into the therapeutic potential of these neuropeptides 18, 19.

Centrally administered AVP or stimulated i.c.v. release of AVP facilitates social recognition in rats, whereas social recognition is impaired in Brattleboro rats (which do not synthesise biologically active AVP) and in rodents administered AVP receptor antagonists centrally 20, 21, 22, 23. Two receptors for AVP are expressed in the brain: Avpr1a and Avpr1b; however, most of the effects of AVP on social memory appear to be mediated primarily via Avpr1a 11, 12, 24. Social recognition is disrupted in *Avpr1a* knockout mice 25 and in rats after central administration of a specific Avpr1a antagonist or antisense oligonucleotides complementary to *Avpr1a* mRNA into either the cerebral ventricle or directly into the lateral septum 21, 26, whereas over‐expression of Avpr1a in the septum markedly enhances social memory in rats 27.

Oxytocin also plays an important role in social memory, although the effects are apparently more complex. Central administration of an oxytocin antagonist blocks social recognition in mice 28 and both oxytocin and oxytocin receptor (*Oxtr*) knockout mice fail to develop social memory for conspecifics 10, 29, 30, with oxytocin given into the brain restoring social recognition in oxytocin knockout mice 28. However, in rats, a low dose of oxytocin given centrally facilitates social recognition in males but not females, whereas central administration of an Oxtr antagonist disrupts social memory in females but not males 31, 32, indicating a possible sex difference for the role of oxytocin in social memory.

The neural circuitry involved in regulating social memory is complex and not fully understood; however, there is evidence to support roles for the lateral septum, bed nucleus of the stria terminalis (BNST), medial amygdala (MeA) and medial preoptic area (MPOA) in oxytocin and AVP‐mediated social recognition 21, 26, 27, 28, 33. Pheromones are detected by receptors on neurones in the vomeronasal organ (VNO) and olfactory epithelium 34. The axons from the VNO and olfactory epithelium neurones project to the accessory olfactory bulb and the main olfactory bulb, respectively, which in turn project to higher brain regions (e.g. the MeA regulating oxytocin and AVP‐mediated social recognition) 34, 35, 36.

Studies have demonstrated that neurones in the MPOA, BNST and MeA are activated by a social encounter in wild‐type mice but not in oxytocin knockout mice and that gene expression for *Oxtr* and activation of Oxtr in the MeA is essential for social recognition in mice 28, 37. Furthermore, local infusion of oxytocin into the MPOA facilitates social memory in rats 33, whereas the effects of AVP in promoting social memory are dependent upon actions via Avpr1a in the lateral septum 21, 26, 27 but not in the MeA 38 or MPOA 33. The MeA projects to the BNST 39 and both these regions provide vasopressinergic innervation to the lateral septum 40, 41, which abundantly expresses Avpr1a 42. Moreover, social interaction has been shown to alter Avpr1a binding in the lateral septum and BNST in rats 43. There are also reciprocal connections between the lateral septum and MPOA and the BNST and MPOA 44, 45.

Although much is known about the effects of prenatal stress on the neuroendocrine regulation of the stress axis 2, 46, less is known about its effects on neuroendocrine regulation of social behaviours. Prenatal stress has been shown to reduce social interaction in male rats and is associated with reduced oxytocin mRNA and oxytocin and AVP immunoreactivity in the PVN and altered Oxtr and Avpr1a binding in the central amygdala 9, 47. Recently, social memory deficits in a habituation–dishabituation paradigm were reported in PNS rats; however, only males were investigated in the study 9. To date, no studies have examined the effects of prenatal stress on social memory in females, nor have any investigated whether any effects are sex‐specific. Hence, in the present study, we investigated whether repeated exposure to an ethologically relevant social stress (to better reflect the types of stress that pregnant women and managed animals are likely to experience) during pregnancy impacts upon social memory in the adult male and female offspring and we further aimed to determine whether any sex differences were evident. It is well established that hypothalamic‐pituitary‐adrenal (HPA) axis responses to stress are greater in PNS rodents 2 and that stress can impact upon cognition 48, 49; however, it is not known whether social memory is impaired in PNS rats under acute stress conditions, which was another aim of the current study. Given the critical role of both Oxtr and Avpr1a in social memory, we further examined whether PNS alters gene expression for *Oxtr* and *Avpr1a* in brain regions known to be involved in regulating social memory. To confirm that any observed differences in social memory were not a result of altered social preference or deficits in olfaction, we tested whether PNS offspring were averse to social encounters and whether they could distinguish between novel and familiar social and nonsocial olfactory cues.

## Materials and methods

### Animals

Female Sprague–Dawley rats were purchased from Charles River (Margate, UK). Unless otherwise specified, rats were group housed (four to six females, three or four males per cage) in open‐top cages and maintained under a 12 : 12 h light/dark cycle (lights on 07.00 h), at 22 ± 2 °C and 55 ± 5% relative humidity with free access to standard 14% protein rodent diet (Harlan Teklad, Derby, UK). All experiments were approved by the local Animal Welfare and Ethical Review Body and were performed in accordance with the UK Animals (Scientific Procedures) Act 1986 and the European Directive (2010/63/EU).

Control and PNS offspring were bred in‐house. Pregnant rats (approximately 12 weeks old) were obtained by overnight mating with sexually experienced males. Pregnancy was confirmed by the presence of a vaginal semen plug the next morning; this was designated as day 1 of pregnancy (parturition expected on day 22). The diet of breeding females was supplemented with 19% protein diet (Harlan Teklad) throughout pregnancy and lactation. Pregnant rats were initially group housed until day 14 of pregnancy, after which they were housed singly.

### Prenatal stress paradigm

Pregnant rats were repeatedly exposed to social stress using a resident–intruder paradigm as described previously 2. Briefly, pregnant ‘intruder’ rats were transferred to an adjacent room and placed in the cage of an unfamiliar lactating ‘resident’ rat (days 2–8 of lactation; different resident each day) for 10 min/day for five consecutive days from days 16–20 of pregnancy. After each episode of stress, rats were returned to their home cage and transferred back to the holding room. Rats were weighed daily during the stress exposure period. Pregnant females that remained undisturbed in their home cages throughout gestation (except for weighing on days 16–20) were used to generate the control offspring. Following parturition, pups remained with their mothers until weaning on postnatal day 23. Following weaning, offspring were housed in same sex groups by litter, under the conditions described above. For each experiment, a maximum of two rats per sex per litter was used per group. For behavioural tests in the offspring, rats were transferred to the behavioural room at least 3 days before start of the experiment.

### Experiment 1: Social preference

At 12 weeks of age, male and female control and PNS rats (n = 9 per group per sex) were tested for social preference using a paradigm adapted from the Crawley test for sociability in mice 50. The test uses same‐sex adult ‘stimulus’ rats placed in clear plastic chamber (150 × 150 × 185 mm) with holes of 8 mm in diameter (23 holes/100 mm^2^), which permits the assessment of motivation for social interaction in the test subjects without the confounding effect of fear of attack from the stimulus rat. Stimulus rats were born in‐house and were approximately 1 week older than the test rats. The stimulus animals were acclimatised to the chambers for 10 min/day for 3 weeks prior to testing. On the day before the test, stimulus rats were weighed to create weight‐matched pairs.

The apparatus consisted of three compartments: two transparent plastic square compartments (each 40 × 40 × 40 cm) connected by doors via a narrow white plastic rectangular compartment (20 × 40 × 40 cm). On the test day, plastic dividing walls were inserted into the apparatus to create three separate compartments. First, the test rat was placed in the middle chamber and allowed to habituate for 3 min. Next, the chamber doors were opened, allowing the rat to freely explore all three chambers (one containing an empty stimulus rat container). After the 10‐min ‘exploration’ phase, the rat was gently guided to the middle chamber and the doors were closed. Meanwhile, the chamber holding the stimulus rat was placed in one of the side compartments and a novel object (50‐ml falcon tube containing a marker pen lid) was placed in the other. The doors were opened again and the test rat was allowed to freely explore the three compartment apparatus for 10 min (‘test’ phase). Rats were tracked during the ‘exploration phase’ and the ‘test phase’ using a ceiling‐mounted infrared camera located directly above the apparatus and the time that rats (nose point, centre point and base of tail point) spent in each compartment was scored automatically using ethovision xt (Noldus, Wageningen, the Netherlands). Additionally, the time spent investigating the novel object and the stimulus rat chamber (defined as active sniffing of the object/stimulus rat chamber) was scored manually using pre‐assigned keystrokes and ethovision xt software.

Testing occurred between 10.00 and 15.00 h (during the light phase). Objects and stimulus animals were randomly assigned to the left or right compartment to control for side preference in the test animals. All of the apparatus was thoroughly cleaned with 70% ethanol between tests. The order in which rats were tested was random (except that males and females were tested on separate days) and the experimenter was blind to the prenatal history of the animals.

### Experiment 2: Social memory under basal conditions

The protocol used to assess social memory was adapted from Engelmann *et al*. 51. On the morning of the test (at approximately 09.00 h), male and female, control and PNS rats (the same rats used in Experiment 1, now 13–14 weeks of age; n = 9 per sex per group) were transferred into clean transparent plastic cages (460 × 240 × 210 mm in size, containing no bedding; one rat per cage) and left undisturbed for 2 h to familiarise them with the test environment. Food and water was available *ad lib*. After 2 h, a same sex juvenile (juvenile A; approximately 21–28 days old) was introduced into cage, and the test rat was allowed to freely investigate the juvenile for 4 min (‘acquisition’ phase). Exploration time, defined as the test rat's nose being within 1 cm of the juvenile and actively sniffing, was recorded. The juveniles were then removed for a pre‐defined lag period or inter‐trial interval (30 min, 1 h, 2 h or 3 h for males; 1, 2 or 3 h for females) based on previous studies that report shorter social memory retention in males compared to females 52. After the lag period, the now ‘familiar’ juvenile A was returned to the adult test rats cage, together with a second novel same‐sex juvenile (juvenile B; also 21–28 days old but from a different litter than juvenile A), for a 4‐min ‘choice’ phase. The time spent investigating the familiar and the novel juveniles was measured. Prior to testing, the juveniles were marked with a marker pen so that they could be easily distinguished. Rats were tested every second day (different sexes on separate days) with the experimenter blinded to the condition of the test animal. One week after the final test day, rats were exposed to a CO_2_ overdose and decapitated. Brains were rapidly removed, frozen on dry ice and then stored at −75 °C until sectioning and processing by *in situ* hybridisation for *Oxtr* and *Avpr1a* mRNA.

### Experiment 3: Social memory under acute stress conditions

Social memory was also tested following acute stress exposure in a separate cohort of control and PNS male and female offspring (aged 20 weeks; n = 9–10 rats per group per sex). For the first part of the experiment, the social memory protocol used was the same as that described for Experiment 2 (above), except that only one lag time was used for each sex and this was selected based on findings from the Experiment 1 above: a 1‐ and 3‐h time lag was used for males and females, respectively. Three days later, the social memory test was repeated using the same adult test rats (with different juveniles); however, this time, all of the rats were exposed to a 30‐min restraint in a transparent plastic ventilated rodent restraint tube (internal diameter: 65 mm), immediately prior to the ‘acquisition’ phase.

### Experiment 4: Effect of acute stress on central *Avpr1a* mRNA expression in PNS females

A separate cohort of 27‐week‐old PNS females (n = 16; eight littermate pairs) were used to test the effect of acute stress exposure on *Avpr1a* mRNA expression in the lateral septum and BNST. One rat from each pair (n = 8) was exposed to 30‐min restraint (as before) and then transferred into a single cage for 3 h to mimic the conditions in the social memory under acute stress experiment. The other rat from each pair (n = 8) was transferred into a single cage for the duration of the experiment (i.e. 3.5 h) and served as a nonrestrained control. Rats were killed by CO_2_ overdose followed by decapitation. Brains were rapidly removed, frozen on dry ice, and then stored at −75 °C until sectioning and processing by *in situ* hybridisation for *Avpr1a* mRNA.

### Experiment 5: Olfactory memory for social and nonsocial odours in control and PNS females

To test olfactory memory for social and nonsocial odours, separate groups of control and PNS female rats (aged 15 weeks; n = 9–10 rats per group per sex) were exposed to spherical wooden beads (diameter 25 mm) that were previously placed for either (i) 1 week in a cage housing two unfamiliar same‐sex conspecifics (social odour) or (ii) 2 days in a sealed zip‐lock bag containing either ground cumin or ground coriander mixed 1 : 1 with aquarium sand (nonsocial odour). Rats were first tested using social odours and, 2 days later, were tested using nonsocial odours. The procedure broadly followed the social memory protocol described above. Rats were separated into clean plastic cages for 2 h and allowed to familiarise themselves with the test environment. Subsequently, rats were allowed to freely explore bead A (impregnated with either a social or nonsocial odour, depending on the test) for 4 min. The bead was disposed of immediately after the first session. After a 3‐h lag time, rats were exposed to two beads: a fresh bead A (with the same odour as before) and a bead B (an unfamiliar odour from the same category; i.e. either a different conspecific odour or a different spice) for a 4‐min ‘choice’ phase. The time spent investigating each bead was measured. The experimenter changed gloves between handling each set of beads and the olfactory cues were presented in a pseudorandom manner, by alternating the odour rats were exposed to first between tests, as well as the location (left or right) of the beads in the cage during the ‘choice’ phase.

In the behaviour experiments, any rat that spent < 5 s in total investigating the stimuli (i.e. object + adult conspecific, juvenile A + B, wooden bead A + B) during the ‘choice’ phase was excluded from the analysis.

### *In situ* hybridisation (ISH)

Brains were cut into 18‐μm coronal sections using a cryostat and thaw‐mounted onto DNase/RNase free Polysine® slides (Thermo Scientific, Waltham, MA, USA) (four sections/slide) and stored at −75 °C until ISH processing. ^35^S‐UTP radiolabelled cRNA antisense probes were used to detect rat *Oxtr* mRNA and *Avpr1a* mRNA. For *Oxtr* mRNA, ^35^S‐UTP labelled sense and antisense riboprobes were synthesised from the linearised pGEM‐7Z vector expressing a 400‐bp cDNA fragment encoding rat *Oxtr* 53. The plasmid was linearised with *Eco*RI and *Bam*HI and transcribed from the SP6 and T7 promoters to synthesise the sense and antisense cRNA probes, respectively. To detect *Avpr1a* mRNA, ^35^S‐UTP labelled sense and antisense riboprobes were synthesised from the linearised pGEM3z vector expressing a 396‐bp cDNA fragment encoding rat *Avpr1a* 42. The plasmid was linearised with *Hin*dIII and *Eco*RI and transcribed from the T7 and SP6 promoters to synthesise the sense and antisense cRNA probes, respectively. ISH was performed as previously described using riboprobes 54, except that overnight hybridisation was performed at 55 °C for both *Oxtr* and *Avpr1a*, and the post‐hybridisation washes (following RNase treatment) were performed as follows: 3 × 60‐min washes in 0.1× SSC at 55 °C for *Oxtr* and 65 °C for *Avpr1a*. Sections were dehydrated in an ascending ethanol series (containing 300 mm ammonium acetate), then dipped in liquid autoradiographic emulsion (Ilford K5; Calumet, Edinburgh, UK) and exposed at 4 °C for 6 or 7 weeks for *Oxtr* mRNA and *Avpr1a* mRNA, respectively. Following exposure, slides were developed, fixed, counterstained with haematoxylin and eosin (for identification of regions of interest) and cover‐slipped with DPX. All sections from the same experiment were processed for a specific mRNA of interest together, except that sections from males and females were processed separately. Sections hybridised with sense probes served as negative controls and showed no signal above background.

### ISH analysis

Regions of interest were identified in consultation with a rat brain atlas 55. *Oxtr* mRNA was analysed in the lateral septum (intermediate part, LSi; bregma +0.84 to +0.24 mm), the BNST (dorsal and posterior to the anterior commissure; bregma −0.6 to −0.96 mm), the MPOA (Bregma 0 to −0.36 mm) and the MeA (Bregma −1.72 to −2.92 mm). *Avpr1a* mRNA was analysed in the anterior lateral septum (aLS: Bregma 1.8–0.72 mm) and posterior lateral septum (pLS: Bregma 0.48 to −0.36 mm) and the BNST (anterior part: Bregma 0.48 to −0.36 mm).

Brightfield photomicrographs were captured using an Eclipse N1 microscope and an Axiocam 105 (Nikon, Tokyo, Japan) colour camera with zen 2012 software (blue edition) (Carl Zeiss, Oberkochen, Germany). The area of each region of interest and the overlying silver grain area were measured using image j, version 1.46h (US National Institutes of Health, Bethesda, MD, USA). Data are expressed as grain area/region of interest area (mm^2^/mm^2^). Background measurements were made over an adjacent area, converted to mm^2^/mm^2^ and subtracted. Bilateral measurements were made from four to eight sections per rat and the group mean ± SEM was calculated from the average values per rat.

### Statistical analysis

Data were analysed using sigmaplot, version 11.0 (Systat Software Inc., Chicago, IL, USA) or minitab, version 17 (2010) (Minitab Inc., State College, PA, USA). For simple comparisons between groups (e.g. mRNA data), a Student's t‐test was used to analyse normally distributed data and a Mann–Whitney U‐test was used to analyse data that were not normally distributed. For behaviour tests comparing two factors, a two‐way anova (acquisition phase) or two‐way repeated measures (RM) anova (choice phase) was used, followed by Student–Newman–Keuls (SNK) multiple comparison tests. For behaviour tests comparing three factors, a three‐way RM anova was used followed by Tukey's pairwise comparison tests. Where the minitab software does not detect an effect of the repeated measures, it automatically reverts to analysing the data by three‐way anova. In each case, P ≤ 0.05 was considered statistically significant.

## Results

### Experiment 1: Social preference

#### Males

**Figure 1:**
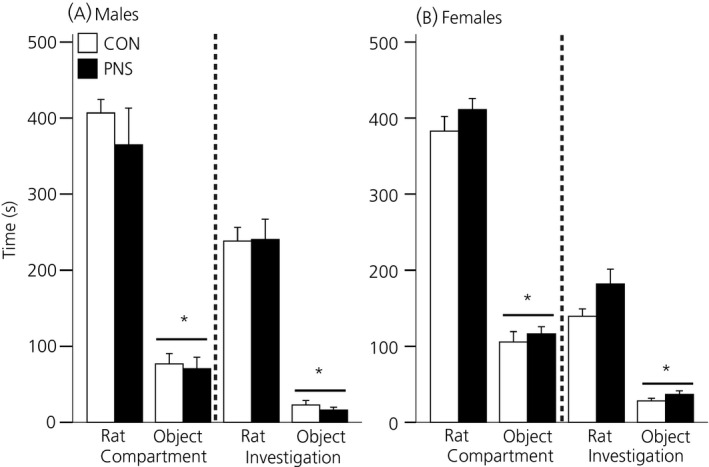
Social preference in control (CON) and prenatally stressed (PNS) rats. Time spent in the compartment containing the stimulus rat or the object (left) and time spent investigating the stimulus rat or the object (right) for control (white bars) and PNS (black bars) (a) males and (b) females. Both males and females demonstrated a strong preference for spending time with the stimulus rat over the object. *P < 0.001 versus rat chamber/rat investigation. Data are the group mean ± SEM (n = 9 rats per group).

Neither the control, nor the PNS males displayed a preference for the left‐ or right‐hand side compartment during the exploration phase, nor were there any significant differences between groups (data not shown). During the test phase, there was a significant effect of the stimuli (i.e. rat or object) on the time spent in a particular compartment (F_1,15_ = 98.37, P < 0.001, two‐way RM anova) and a significant effect of the stimuli on investigation time (F_1,15_ = 201.68, P < 0.001, two‐way RM anova). Males spent significantly more time in the compartment housing the stimulus rat compared to the compartment containing the object (control, P < 0.001; PNS, P < 0.001, SNK test) (Fig. 1 a, left) and spent significantly more time investigating the stimulus rat compared to the object (P < 0.001 for control and PNS males, SNK test) (Fig. 1 a, right). There were no differences between the control and PNS males for either compartment time or investigation time and no prenatal experience × compartment interactions were detected.

#### Females

Neither the control, nor the PNS females exhibited a preference for the left‐ or right‐hand side compartment during the exploration phase, nor were there any significant differences between groups (data not shown). During the test phase, there was a significant effect of the stimulus (i.e. rat or object) on the time spent in a particular compartment (F_1,16_ = 217.62, P < 0.001, two‐way RM anova) and a significant effect of the stimulus (F_1,16_ = 110.68, P < 0.001, two‐way RM anova) on investigation time. Females spent significantly more time in the compartment housing the rat compared to the compartment containing the object (P < 0.001 for control and PNS females, SNK test) (Fig. 1 b, left) and spent significantly more time investigating the stimulus rat compared to the object (P < 0.001 for control and PNS females, SNK test) (Fig. 1 b, right). However, there were no differences between the control and PNS females for either compartment time or investigation time and no prenatal experience × compartment interactions were detected.

#### Sex differences

There were no significant differences between males and females in the time spent in a particular compartment (three‐way anova). However, there was a significant effect of sex (F_1,62_ = 11.52, P = 0.001) and a significant interaction between sex and stimulus on the time spent investigating the stimuli (F_1,62_ = 323.19, P < 0.001). Male rats spent significantly more time investigating the stimulus rat than females (P < 0.001, Tukey's test).

### Experiment 2: Social memory under basal conditions

There were no differences between the control and PNS groups in the time spent investigating juvenile A during the acquisition phase for either sex. However, there was a significant effect of sex (F_1,120_ = 141.16, P < 0.001; two‐way anova), with both control (121.4 ± 3.3 s) and PNS (118.0 ± 3.9 s) males spending significantly more time (P < 0.001, SNK test) investigating juvenile A than the control (75.7 ± 4.9 s) and PNS females (64.9 ± 4.9 s) during the acquisition phase.

#### Males

**Figure 2:**
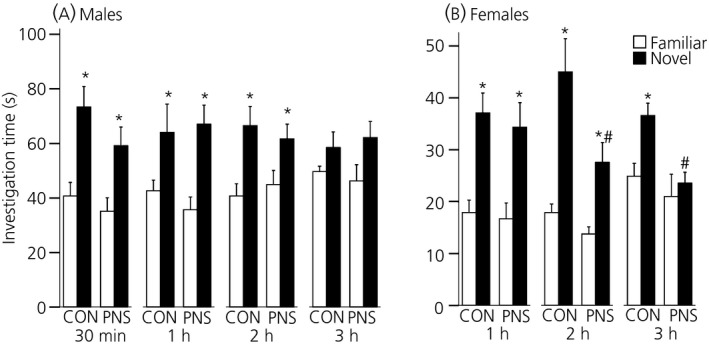
Social memory under basal conditions in control (CON) and prenatally stressed (PNS) rats. Time spent investigating the familiar (white bars) versus the novel (black bars) juvenile under basal conditions following various inter‐trial intervals in (a) males and (b) females. Both control and PNS males demonstrated a preference for the novel juvenile following 30‐min, 1‐ and 2‐h inter‐trial intervals, but not after the 3‐h interval. Control and PNS females demonstrated a preference for the novel juvenile following the 1‐ and 2‐h inter‐trial interval but, after the 3‐h interval, whereas control females still demonstrate a preference for the novel juvenile, PNS females do not. *P < 0.05 familiar versus novel; #P < 0.005 control versus PNS. Data are the group mean ± SEM (n = 9 rats per group).

There were no differences between the control and PNS males in the time spent investigating the juveniles during the ‘choice’ phase; however, there was a significant effect of novelty on the time spent investigating the juveniles after an inter‐trial interval of 30 min (F_1,16_ = 15.8, P < 0.001; two‐way RM anova), 1 h (F_1,15_ = 12.78, P = 0.003) and 2 h (F_1,16_ = 11.31, P = 0.004) but not after the 3‐h interval (Fig. 2 a). For the 30‐min, and 1‐ and 2‐h intervals, F1 control males (30 min, P = 0.005; 1 h, P = 0.04; 2 h, P = 0.011; SNK test) and F1 PNS males (30 min, P = 0.03; 1 h, P = 0.008; 2 h, P = 0.04; SNK test) spent significantly more time investigating the novel juvenile compared to the familiar juvenile (Fig. 2 a); however, after the 3‐h interval, the male rats demonstrated no preference for the familiar versus the novel juvenile and there was no significant difference between the control and PNS rats (Fig. 2 a).

#### Females

There was a significant effect of novelty but not prenatal experience on the time spent investigating the juveniles after an inter‐trial interval of 1 h (F_1,16_ = 50.27, P < 0.001; two‐way RM anova) and 2 h (F_1,16_ = 27.2, P < 0.001), with both control (1 h, P < 0.001; 2 h, P < 0.001; SNK test) and PNS (1 h, P < 0.001; 2 h, P = 0.025; SNK test) females spending significantly more time investigating the novel juvenile compared to the familiar juvenile (Fig. 2 b). However, after the 3‐h inter‐trial interval, there was a significant effect of both prenatal experience (F_1,16_ = 6.38, P = 0.02; two‐way RM anova) and novelty (F_1,16_ = 8.23, P = 0.012), with the control (P = 0.004, SNK test) but not the PNS females (P = 0.48; SNK test) exhibiting a preference for the novel juvenile (Fig. 2 b). Furthermore, after the 2‐h (P = 0.003; SNK test) and 3‐h (P = 0.004; SNK test) inter‐trial intervals, PNS females spent significantly less time investigating the novel juvenile compared to the control females (Fig. 2 b).

#### Sex differences

During the choice phase, there was a significant overall effect of sex (1 h: F_1,32_ = 36.49, P < 0.001; 2 h: F_1,64_ = 64.13, P < 0.001; 3 h: F_1,32_ = 82.55, P < 0.001; three‐way RM anova), with both control and PNS males spending more time investigating the familiar (P < 0.001, Tukey's test) and novel (P < 0.001, Tukey's test) juveniles than the female rats (Fig. 2).

### Central *Oxtr* and *Avpr1a* mRNA expression under basal conditions

#### Males

**Figure 3:**
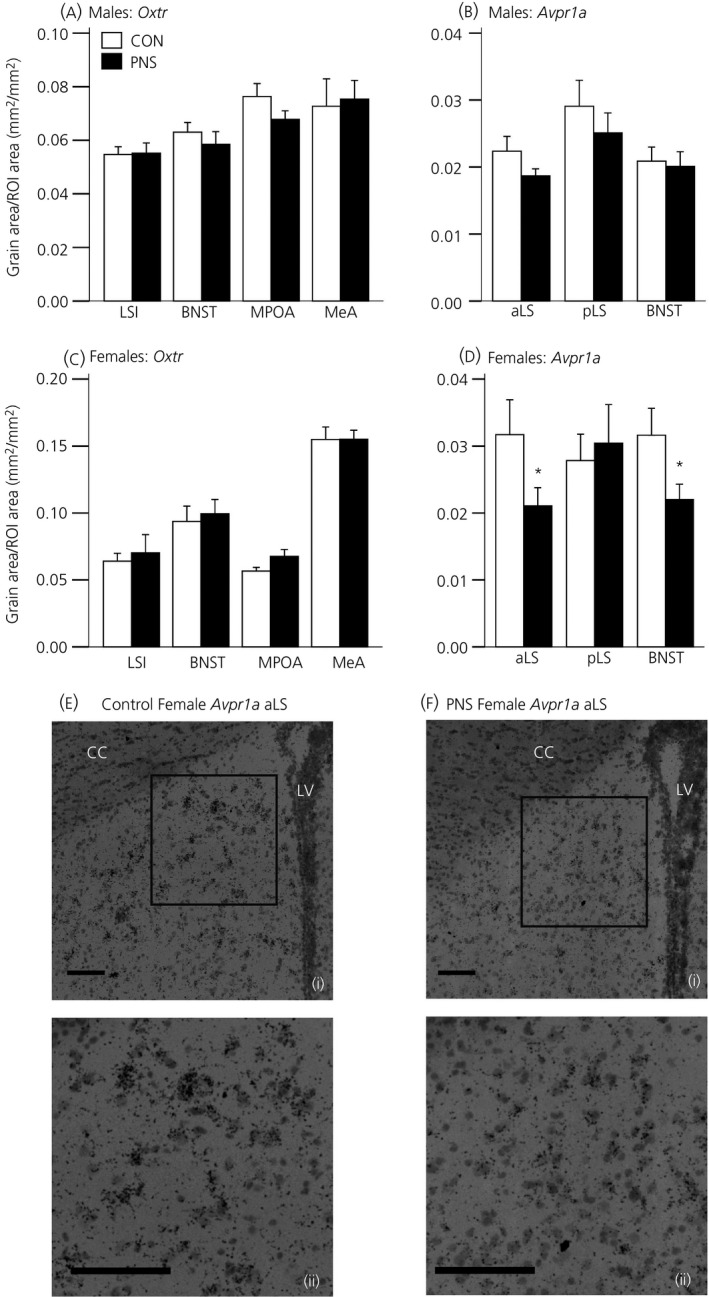
Central *Oxtr* and *Avpr1a* mRNA expression under basal conditions in control (CON) and prenatally stressed (PNS) rats. Quantification of *Oxtr* mRNA expression in the intermediate lateral septum (LSI), medial preoptic area (MPOA), bed nucleus of stria terminalis (BNST) and medial amygdala (MeA) in control (CON; white bars) and prenatally stressed (PNS; black bars) (a) males and (c) females. *Avpr1a* mRNA expression in the anterior lateral septum (aLS), posterior lateral septum (pLS) and BNST in (b) males and (d) females. *P < 0.04 versus control group. Representative photomicrographs of *Avpr1a* mRNA expression in the aLS from a (e) control female and a (f) PNS female under (i) low and (ii) high power. LV, lateral ventricle; cc, corpus callosum. Scale bars = 100 μm. Data are the group mean ± SEM (n = 9 rats per group for control males/females and PNS males; n = 8 rats per group for PNS females). ROI, region of interest.

There were no significant differences in *Oxtr* (Fig. 3 a) or *Avpr1a* (Fig. 3 b) mRNA expression between control and PNS males in any of the brain regions examined.

#### Females

No differences were detected in *Oxtr* mRNA expression in the LSi, MPOA, BNST and MeA between control and PNS females (Fig. 3 c). *Avpr1a* mRNA expression was significantly lower in the aLS (U_15_ = 17, Z = −1.83, P = 0.037; Mann–Whitney U‐test) and in the BNST (t_15_ = 2.01, P = 0.032; Student's t‐test) of the PNS females compared to the control females, although there were no group differences detected in the pLS (Fig. 3 d).

### Experiment 3: Effect of acute stress on social memory

#### Males

**Figure 4:**
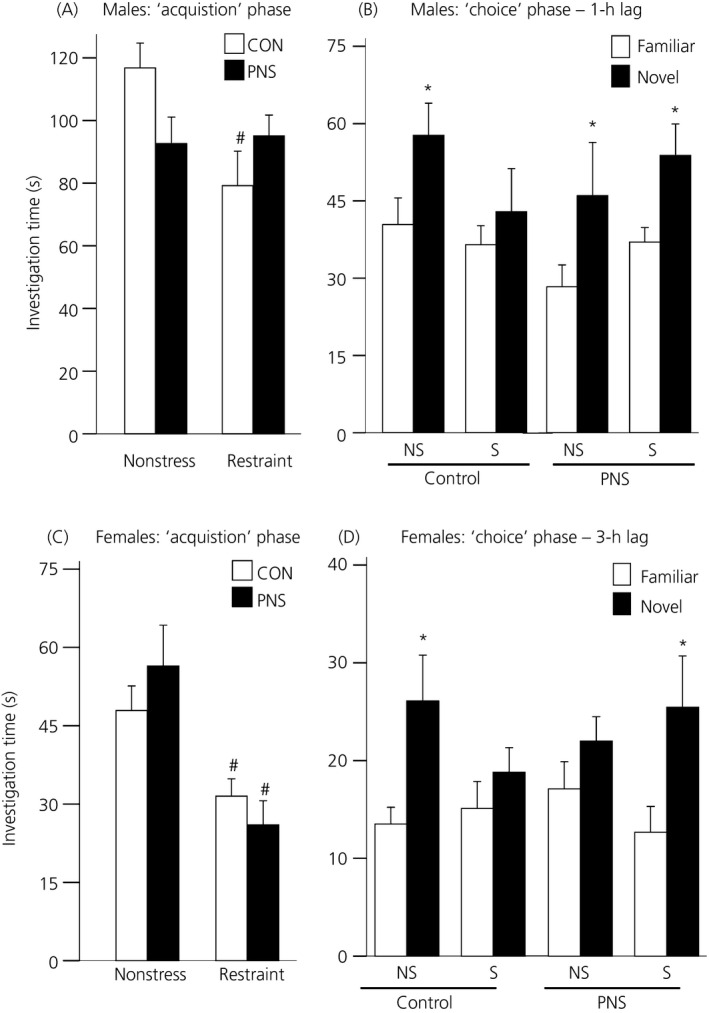
Effect of acute stress on sociability and social memory in control (CON) and prenatally stressed (PNS) rats. Time spent investigating juvenile A during the acquisition phase under nonstress and acute stress (30‐min restraint) conditions in control (CON; white bars) and prenatally stressed (PNS; black bars) (a) males and (c) females. Control but not PNS males displayed a decrease in the time spent investigating juvenile A immediately following stress (i.e. during the acquisition phase), whereas both control and PNS females displayed a decrease in the time spent investigating juvenile A during the acquisition phase. Time spent investigating the familiar (white bars) versus the novel (black bars) juvenile under nonstress (NS) and stress (S) conditions during the ‘choice’ phase in (b) males after a 1‐h inter‐trial interval and (d) females after a 3‐h inter‐trial interval. Both control and PNS males demonstrated a preference for the novel juvenile under nonstress conditions; however, following acute stress, only the PNS males exhibited a preference for the novel juvenile. Control but not PNS females displayed a preference for the novel juvenile under nonstress conditions; however, under acute stress conditions, control females no longer displayed a preference for the novel juvenile, whereas PNS females did. #P < 0.03 versus nonstress group with same prenatal treatment; *P ≤ 0.05 known versus novel juvenile within the same group and treatment. Data are the group mean ± SEM (n = 10 rats per group for control males/females and PNS males; n = 9 rats per group for PNS females).

Restraint had a significant effect on the time the male rats spent investigating juvenile A during the acquisition phase (F_1,18_ = 4.35, P = 0.05, three‐way RM anova) (Fig. 4 a). There was also a significant interaction between prenatal experience and acute stress (F_1,18_ = 5.68, P = 0.028), with restraint exposure significantly decreasing investigation time in the control (P = 0.025, Tukey's test) but not the PNS males (Fig. 4 b).

There was a significant effect of novelty (F_1,18_ = 13.94, P < 0.001, three‐way RM anova) and a significant prenatal experience × acute stress interaction (F_1,18_ = 4.66, P = 0.045) on the time that male rats spent investigating the familiar and novel juveniles with and without stress. Under nonstress conditions, both control and PNS males spent significantly more time investigating the novel juvenile than the familiar juvenile (control: P = 0.05, PNS: P = 0.02, Tukey's test) (Fig. 4 b); however, after restraint, only PNS males exhibited a preference for the novel juvenile (P = 0.015, Tukey test) (Fig. 4 b).

#### Females

Restraint significantly decreased the time both the control and PNS females spent investigating juvenile A during the acquisition phase (F_1,17_ = 23.53, P < 0.001, three‐way RM anova) (Fig. 4 c).

There was a significant effect of novelty (F_1,17_ = 24.93, P < 0.001; three‐way RM anova) and a significant three‐way interaction between prenatal experience × acute stress × novelty (F_1,17_ = 4.94, P = 0.04) on the time that female rats spent investigating the familiar and novel juveniles with and without stress during the choice phase. Under nonstress conditions, control females spent significantly more time investigating the novel juvenile (P = 0.005, Tukey's test) (Fig. 4 d); however, following exposure to restraint, the control females no longer discriminated between the familiar and the novel juvenile. PNS females exhibited no preference for either juvenile under nonstress conditions but, following stress exposure, they spent significantly more time investigating the novel juvenile compared to the familiar juvenile (P = 0.024, Tukey's test) (Fig. 4 d).

### Experiment 4: Effect of acute stress on *Avpr1a* mRNA expression in the lateral septum and BNST

**Figure 5:**
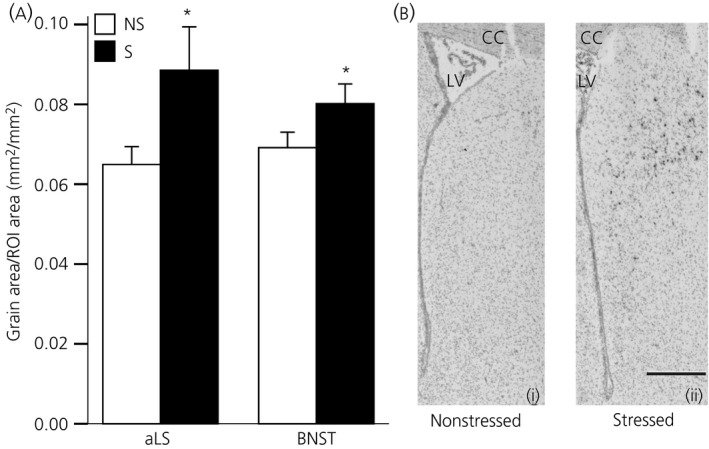
Effect of acute stress on central *Avpr1a* mRNA expression in prenatally stressed (PNS) females. (a) Quantification of *Avpr1a* mRNA expression in the anterior lateral septum (aLS) and bed nucleus of the stria terminalis (BNST) in nonstressed (ns; white bars) or stressed (restraint; black bars) PNS females. PNS females exposed to acute stress had significantly greater *Avpr1a* mRNA expression in both regions compared to nonstressed PNS females. *P ≤ 0.05 versus nonstress group. (b) Representative photomicrographs of *Avpr1a* mRNA expression in the aLS from a (i) nonstressed, and (ii) restrained PNS female. LV, lateral ventricle; cc, corpus callosum. Scale bar = 500 μm. Data are the group mean ± SEM (n = 8 rats per group). ROI, region of interest.

*Avpr1a* mRNA expression was significantly greater in both the aLS (U_16_ = 50.5, Z = 1.94, P = 0.026, Mann–Whitney U‐test) and in the BNST (t_14_ = −1.76, P = 0.05; Student's t‐test) in PNS female rats exposed to 30 min restraint compared to the nonstressed PNS females (Fig. 5).

### Experiment 5: Olfactory memory for social and nonsocial odours

There was no effect of prenatal experience on the investigation times during the acquisition phase for either the social (CON, 27.2 ± 3.9 s; PNS, 27.3 ± 6.2 s) or the nonsocial odours (CON, 40.3 ± 9.7 s; PNS, 26.9 ± 6.1).

**Figure 6:**
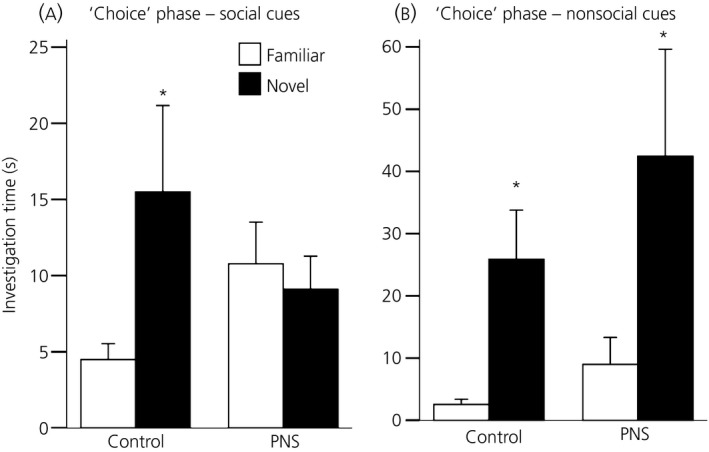
Olfactory memory for social and nonsocial odours in control (CON) and prenatally stressed (PNS) female rats. Time spent investigating beads impregnated with familiar (white bars) and novel (black bars) (a) social odours and (b) nonsocial odours in control (CON) and prenatally stressed (PNS) females after a 3‐h inter‐trial interval. Control but not PNS females demonstrated a preference for the novel social odour. Both control and PNS females displayed a preference for novel nonsocial odours. *P ≤ 0.03 familiar versus novel within same group. Data are the group mean ± SEM (n = 8 control and n = 9 PNS rats per group for the social odour experiment; n = 9 rats per group for the nonsocial odour experiment).

There was a significant prenatal experience × novelty interaction during the social odour choice phase (F_1,15_ = 4.55, P = 0.05; two‐way RM anova). Although control females showed a preference for the novel social odour over the familiar odour (P = 0.023, SNK test), PNS females did not (P = 0.688, SNK test) (Fig. 6 a).

During the choice phase for the nonsocial odours, there was a significant effect of novelty (F_1,16_ = 8.27, P = 0.01; two‐way RM anova). Investigation times for the novel odours were significantly greater than for the familiar odours in both the control (P = 0.005; SNK test) and PNS (P = 0.03; SNK test) females (Fig. 6 b), with no differences between the groups.

## Discussion

In the present study, we report a sex‐specific deficit in social memory in female PNS rats, which was associated with reduced *Avpr1a* mRNA expression in brain regions known to be critically involved in regulating social recognition. By contrast, acute stress increased central *Avpr1a* mRNA expression and was associated with facilitated social memory in the PNS females.

Under basal conditions, PNS females displayed impaired social memory over a 3‐h period; however, social memory in PNS males was similar to that observed in control males. The deficit in social memory observed in the PNS females is unlikely to reflect reduced sociability or an aversion to social interaction because the investigation times were similar in control and PNS females during the social preference test and during the acquisition phase of the social memory test. This rules out the possibility that the PNS females failed to remember the familiar juvenile after 3 h because they had interacted with it less during the acquisition phase. Similarly, we can exclude neophobia in PNS females as an underlying factor in explaining the difference in social recognition because the investigation times for the novel juvenile were similar in control and PNS females at the 1‐h time‐point. Other studies have previously reported altered (i.e. more aggressive encounters) or reduced social interaction in different rat models of prenatal stress 9, 47; however, this discrepancy is likely explained by the different conditions under which the sociability tests were performed. Given that increased anxiety‐like behaviour and fearfulness have been reported in PNS rats 2, 56, 57, 58, in the present study, stimulus rats were confined within a chamber, allowing rats to be investigated without fear of attack and permitting an assessment of motivation for social interaction at the same time as minimising the confounding effect of enhanced fear and/or anxiety increasing avoidance or defensive withdrawal behaviour 58. Previous studies investigated social interaction in freely moving dyads, which is likely to be more anxiogenic than the test applied in the present study, especially given the higher levels of anxiety‐like behaviour reported in the offspring 9. Indeed, increased anxiety‐like behaviour is associated with increased corticotrophin‐releasing hormone release in the amygdala 58, 59 and central administration of corticotrophin‐releasing hormone reduces social interaction in rats 60.

The central oxytocin and AVP systems play critical roles in processing social memory cues 11, 12. Accordingly, we found a marked reduction in gene expression for *Avpr1a* in the lateral septum and the BNST in the PNS females (which exhibited a social memory deficit) compared to control females but not in the male PNS rats (where social memory did not differ from controls). Further studies are required to establish whether this reduced gene expression translates to a reduction in Avpr1a abundance or binding; however, given the vital role that AVP plays in the lateral septum in processing and/or retrieval of social memory 21, 26, 27, 61, reduced sites for AVP action may contribute to impaired social recognition in the PNS females. In support, impaired social memory in adult male rats exposed to stress in early postnatal life evidently involves a reduced activation of Avpr1a in the lateral septum as a result of blunted intraseptal AVP release 62. Whether AVP release in the septum is altered during social memory acquisition in the PNS females used in the present study remains to be determined. By contrast to the findings for social memory, reduced *Avpr1a* mRNA expression in the PNS females was not associated with altered social preference; however, this was expected given that central administration of either AVP or an Avpr1a antagonist has no effect on social preference in female rats 63.

We did not find any differences in *Oxtr* mRNA in any of the regions examined; however, reduced expression of Avpr1a in the lateral septum and BNST may also impact upon oxytocin‐mediated social memory processes, given that oxytocin evidently exerts some of its effects on sociability, social recognition and social communication via Avpr1a, rather than Oxtr 64, 65. Moreover, oxytocin signalling may be impaired in PNS rats as a result of reduced oxytocin synthesis in the PVN, as previously reported in other models of prenatal stress 9, 47.

In the present study, we found a sex difference in social memory in the PNS rats with control and PNS males performing similarly in the social recognition task, whereas only the control females could discriminate between the familiar and novel juveniles after a 3‐h interval. Other studies have reported social memory deficits in the male offspring (females were not tested) of dams exposed to repeated restraint during late pregnancy 9, suggesting that males or females may be more susceptible to the effects of prenatal stress exposure depending on the paradigm utilised. The mechanism underlying the sex difference in social recognition in the PNS rats reported in the present study is not known, although it may involve differences in the actions of oestrogen, given that oestrogen facilitates social recognition, primarily via actions on oestrogen receptor‐α (Esr1) 66, 67. Importantly, the central expression of *Avpr1a* mRNA is evidently up‐regulated by oestrogen 68, 69, and thus reduced sensitivity to oestrogen may explain the reduced *Avpr1a* mRNA expression in PNS females and hence impaired social recognition. Testosterone has also been implicated in regulating social memory in male rodents through actions on vasopressinergic signalling 70. Castration disrupts social memory (although this is dependent on the timing of the testing) 70, reduces AVP immunoreactivity in the BNST and MeA 71 and reduces Avpr1a binding in the medial preoptic nucleus, the MPOA and the BNST 72. These effects of testosterone are evidently mediated, at least in part, via aromatisation to oestradiol 73, 74. Although we do not know whether aromatase expression is altered by PNS exposure, we recently reported significantly greater levels of circulating testosterone in male PNS rats 46, which may help explain why social memory and central *Avpr1a* mRNA was unaffected by prenatal stress in males.

Acute stress exposure prior to social recognition testing impaired social memory in both male and female control rats; however, it had no effect in the PNS males and, intriguingly, facilitated social memory acquisition and/or retrieval in the PNS females. The social memory deficits observed in the control rats may result from the effect of acute stress in decreasing the investigation time of the first juvenile during the acquisition phase. However, although this may help to explain why social memory is impaired in the control but not the PNS males, it does not explain why the PNS females outperform the control females, despite similar investigation times during the acquisition phase. *Avpr1a* mRNA expression was increased in the lateral septum and BNST following exposure to acute stress exposure in the PNS females, supporting a role for increased AVP signalling in these brain regions with respect to facilitating social recognition. In accordance, over‐expression of *Avpr1a* in the lateral septum using viral vectors enhances social memory in male rats 27. The mechanism underlying increased *Avpr1a* transcription is not clear; however, it likely involves regulation by glucocorticoids because adrenalectomy reduces (whereas glucocorticoid administration increases) Avpr1a binding and *Avpr1a* mRNA in the septum and BNST 75. We have previously demonstrated that the female offspring of rats exposed to the same social stress as used in the present study display markedly greater HPA axis responses to restraint 2, and so it is possible that greater restraint‐induced corticosterone secretion in the PNS females led to increased *Avpr1a* mRNA and hence Avpr1a expression, which in turn facilitated social memory in the social recognition task. Acute stress exposure increases local oxytocin and vasopressin release within the septum and hypothalamus 76. Whether restraint differentially affects the pattern and/or amount of these neuropeptides released in the brain in control and PNS rats, and whether this may explain differences in social memory following stress exposure, is not known but warrants further investigation.

Prenatal stress is often considered detrimental to the offspring, with exposure to excessive stress during development increasing stress vulnerability in later life. However, an alternative view is that the programmed phenotypes are adaptive, preparing the offspring to cope in a sub‐optimal postnatal environment 77. Under this hypothesis, signalling to the foetuses *in utero* that they are to be born into a stressful environment (e.g. as a result of increased predation, low food availability, increased social competition) induces adaptations to promote survival, which would be beneficial in evolutionary terms. For example, heightened anxiety and stress responsiveness would be expected to increase vigilance and risk aversion, whereas a ‘thrifty’ phenotype triggers metabolic adaptations to aid survival when resources are in short supply 78. However, when there is a ‘mismatch’ between the predicted and actual postnatal development, phenotypic adaptations triggered by environmental signals during early development may be maladaptive and result in adverse effects on future health. In the present study, when there was a match between the predicted and actual postnatal environment (i.e. prenatal stress and adulthood stress), the PNS rats coped better than controls because social memory was not affected by stress in PNS males and was facilitated in PNS females. The mismatch between the pre‐ and postnatal environment in control rats had a negative effect on social memory in both sexes. In support, a recent study demonstrated that mice exposed to stress in early postnatal life display increased anxiety and reduced social interaction when there is a mismatch between the environment in early life and adulthood; however, mice reared under matched adverse conditions behave similarly to those reared in a matched positive environment 79. Taken together with the present data, these findings suggest that the effects of adverse early‐life experiences are more pronounced when there is a mismatch between the early‐ and later‐life environment.

The deficit in social recognition in PNS females does also not appear to be a result of impaired olfaction in these animals or an inability to form olfactory memories of any type because the PNS females, similar to the control females, could discriminate between nonsocial odours after a 3‐h lag time. Rather, the data from the social odour test suggest that the deficit in processing olfactory cues is specific for social odours and not a result of failure to recognise and/or process and retrieve memories for all olfactory cues. Indeed, social and nonsocial olfactory odours are processed through divergent brain pathways 80. It would appear unlikely that altered oxytocin signalling in the olfactory bulb underlies impaired social recognition in the PNS females, given that infusion of an Oxtr antagonist into the olfactory bulb does not affect social recognition in rats 81 or mice 28. Nonetheless, we cannot rule out the possibility of altered AVP signalling in the olfactory bulb, despite inconsistent evidence on the significance of the olfactory bulb AVP system in social recognition 81, 82.

In summary, social memory is impaired in PNS females; however, this does not appear to be a result of reduced sociability or impaired olfaction because PNS females interact with conspecifics to a similar extent as controls and can discriminate between different nonsocial odours. Rather, the effect on social memory appears to be specific to social odours and is associated with reduced central *Avpr1a* mRNA expression. Under stress conditions, social memory is facilitated in PNS females and is associated with an up‐regulation of central *Avpr1a* mRNA expression. Although further studies are required to establish causality, when taken together, these data support a role for altered signalling via central Avpr1a with respect to the sex‐specific effects of prenatal stress on social memory in the offspring. The preservation of social memory under stress conditions in male and female PNS rats but not controls further suggests an adaptive role for foetal programming in aiding PNS offspring to cope with stressful situations in later life. However, problems are likely to emerge when there is a mismatch between the predicted (based on *in utero* signals) and the actual postnatal environment.

Deficits in social behaviour are evident in neurodevelopmental disorders, such as schizophrenia and autism, and polymorphisms in the 5′‐flanking region of the human *Avpr1a* gene have been linked to an increased susceptibility to autism 83 and lower scores in a facial affect recognition task in patients with schizophrenia 84. Moreover, the negative effect of childhood adversity on social integration and social attachment in adulthood is greater in humans with a polymorphism in the *Avpr1a* promoter 85. Taken together, these data suggest that variation in the *Avpr1a* gene contributes to deficits in social behaviour and that early‐life stress can influence this interaction. Thus, further understanding of how stress in early life influences *Avpr1a* gene expression in the brain could lead to novel intervention strategies for neurodevelopmental disorders associated with dysfunctional social behaviours.
